# Measurement of table feed speed in modern CT

**DOI:** 10.1120/jacmp.v15i3.4703

**Published:** 2014-05-08

**Authors:** Atsushi Fukuda, Pei‐Jan P. Lin, Kosuke Matsubara, Tosiaki Miyati

**Affiliations:** ^1^ Department of Radiology Shiga Medical Center for Children Moriyama Shiga Japan; ^2^ Division of Health Sciences Graduate School of Medical Sciences, Kanazawa University Kanazawa Ishikawa Japan; ^3^ Department of Radiology Virginia Commonwealth University Medical Center Richmond VA USA

**Keywords:** CT scanner, table feed speed, gantry rotation time, solid‐state detector, computed radiography

## Abstract

The purpose of this study was to develop and evaluate a noninvasive method to assess table feed speed (mm's) in modern commercial computed tomography (CT) systems. The table feed (mm/rotation) was measured at selected nominal table feed speeds, given as low (26.67 mm/s), intermediate (48.00 mm/s), and high (64.00 mm/s), by utilizing a computed radiography (CR) cassette installed with a photostimulable phosphor plate. The cassette was placed on the examination table to travel through the isocenter longitudinally, with a total scan length of over 430 mm. The distance travelled was employed to determine the total table feed length. To calculate the table feed speed, gantry rotation time was measured concurrently at a preselected nominal rotation time of 750 ms. Upon completion of data acquisition, the table feed and gantry rotation time were analyzed and used to calculate the actual table feed speed (mm/s). Under the low table feed speed setting, the table feed speed was found to be 26.67 mm/s. Similarly, under the intermediate and high table feed speed settings, the table feed speed was found to be 48.10 and 64.07 mm/s, respectively. Measurements of the table feed speed can be accomplished with a CR system and solid‐state detector, and the table feed speed results were in excellent agreement with the nominal preset values.

PACS number: 87.57.Q‐

## I. INTRODUCTION

With the advent of slip ring technology, helical scanning computed tomography (CT) was introduced in 1988.^(12)^ Helical scan technology, utilizing continuous gantry rotation and table feed, provided for longer anatomic coverage in a shorter time.

Accurate control of the table feed speed is one of the important factors in modern CT system because scan pitch is a key factor in coronary CT angiography.^3^, ^4^ High‐pitch (P=3.2) CT angiography has been recently developed, and this technique requires a table feed speed of up to 430 mm/s.^(4)^ Because scan pitch is defined as the ratio of the table feed per gantry rotation to the total nominal scan width,^(5)^ a comprehensive quality assurance (QA) program should include assessing not only the beam width, but also the table feed speed and gantry rotation time.

While the table feed speed is an important scan parameter of any CT scanner, the accuracy and precision of the table feed speed are not necessarily known to medical physicists, whose responsibilities may include the QA of CT scanners. The American Association of Physicists in Medicine (AAPM) published two reports, Report 39^(6)^ and Report 83,^(7)^ on the QA of CT scanners which evaluate specifications and test procedures. Both reports included evaluation of performance of electromechanical components such as table to gantry alignment, table vertical and longitudinal digital indicators, radiation dose profiles, and the X‐ray‐generator kVp accuracy. While the table feed speed was not explicitly included in these reports, the authors opine that verification of the table feed speed is essential for the proper assessment of a modern CT system. To the best of our knowledge, no published methods of measuring the table feed speed are available.

To determine the table feed speed, it is necessary to measure the gantry rotation time. Fukuda et al.^(8)^ reported a simple noninvasive technique to measure the rotation time using two different types of solid‐state detectors. On the basis of their investigation, we considered that a computed radiography (CR) system and solid‐state detector would allow accurate measurement of the table feed speed. This study aims to provide a simple noninvasive approach to assess table feed speed in a modern commercial CT system.

## II. MATERIALS AND METHODS

### A. CT scanner, CR system, and solid‐state detector system

A 4‐channel multidetector CT scanner was employed for this study. The scan parameters employed were as follows: 1) tube potential, 80 kVp; 2) tube current, 10 mA; 3) total collimation width, 32.0mm(8.0mm×4−channel);4) nominal table feed, 20.0, 36.0, or 48.0 mm/rotation (with nominal table feed speed, 26.67, 48.00, or 64.00 mm/s); 5) nominal gantry rotation time, 750 ms; and 6) nominal scan pitch, 0.625, 1.125, or 1.500.

A CR cassette (354 mm×430 mm) installed with a photostimulable phosphor plate (Imaging Plate Cassette Type CC and IP ST‐VI; FUJIFILM Holdings Corporation, Tokyo, Japan) was employed for this study. Thus, the maximum imaging length available was 430 mm for the measurement of tabletop travel. This CR system has three “exposure data recognizer” (EDR) modes: “auto,” “semi,” and “fixed.” The fixed mode was employed to avoid any raw data manipulation by CR system software. Furthermore, the CR images were processed in “fixed EDR” mode, with latitude=4and sensitivity=5using the AVE4.0 test menu, as described by Liu et al.^(9)^

The Xi solid‐state detector (Unfors Raysafe, Billdal, Sweden), which is designed for application in conventional radiography and fluoroscopy,^(10)^ was employed for the measurement of gantry rotation time. As such, the Xi detector is backed with a lead support to limit the detection of backscatter. The signal obtained is sent to a laptop computer wirelessly via Bluetooth (Bluetooth SIG, www.bluetooth.com), and data analysis is handled by the “Xi View” software.

### B. Measurement of table feed per rotation and gantry rotation time

**Figure 1:**
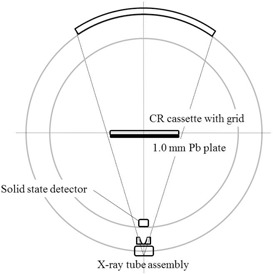
Experimental arrangement for measurements of table feed per rotation and gantry rotation time. The CR cassette was placed on the examination table on a 1 mm thick lead plate to travel through the isocenter longitudinally for the measurement of table feed per rotation under the helical scan mode. Concurrently, the solid state detector was positioned on the inner bottom of the gantry cover for gantry rotation time measurements.

The CR cassette in conjunction with an antiscatter grid (8:1, 60 l/cm, and focus distance 100 cm; Mitaya Manufacturing Corporation, Tokyo, Japan) was placed on the examination table top on a 1.0 mm lead plate to pass longitudinally through the isocenter, as shown in Fig. 1. Although the CR cassette is installed with a 0.13 mm thick lead support, an additional 1.0 mm thick lead sheet was added to minimize the possibility of radiation reaching the photostimulable phosphor plate from the back side of the cassette.

A total scan length of over 430 mm was performed to acquire a helical CT radiation profile. The photostimulable phosphor plate was processed as described above, and the data were exported to a personal computer and analyzed with ImageJ software.^(11)^

While the helical CT radiation profile was being obtained, the Xi detector was positioned face down on the gantry cover, as shown in Fig. 1. Because the Xi detector is backed with a lead support to shield it from backscatter, the detector can measure only the primary radiation as the X‐ray tube passes by the detector at the bottom of the gantry where the detector is located. After the data were acquired, the peak times were determined with the “Xi View” software, and the gantry rotation time was determined as the time between two successive signal peaks.^(8)^

## III. RESULTS

**Figure 2:**
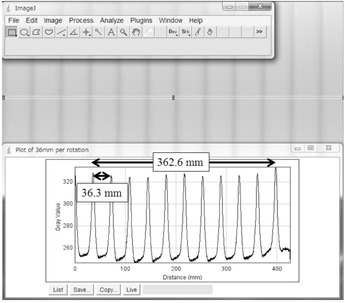
Illustration of the radiation dose profile and table feed distance under the intermediate table feed setting. The CR cassette located at the isocenter was scanned with a nominal gantry rotation time of 750 ms and nominal table feed per rotation of 36.0 mm/rotation, with X‐ray settings of 80 kVp, 10 mA, 32 mm total collimation width. The table feed length over ten gantry rotations is 362.6 mm and the measured table feed per rotation is 36.3 mm/rotation.

Figure 2 depicts the radiation dose profile from the CR cassette as it was scanned using a nominal scan pitch of 1.125 and nominal table feed of 36.0 mm/rotation. This figure includes ten complete radiation profiles. A feed length of 362.6 mm was measured over ten gantry rotations using the plot profile function provided in the ImageJ software. Thus, the actual table feed was determined to be 36.3 mm/rotation.

Similarly, the actual table feed was determined to be 20.1 and 48.3 mm/rotation under the nominal table feed of 20.0 and 48.0 mm/rotation, respectively.

**Figure 3:**
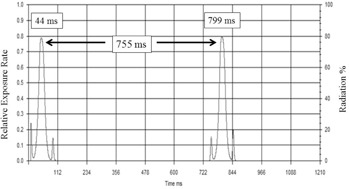
Illustration of the detector output signals/peaks using an Xi detector for measurement of the gantry rotation time. The Xi detector was located on the inner gantry cover, and the measurements were conducted with a nominal gantry rotation time of 750 ms. The two peaks are registered at 44 and 799 ms, respectively. The measured gantry rotation time is 755 ms.

The dose rate profile obtained from the Xi detector, after processing with the “Xi View” software, is shown in Fig. 3. There are two peaks located at rotation times of 44 and 799 ms, respectively. Therefore, the gantry rotation time is determined to be 755 ms.

Table 1 shows the comparison between the nominal preset values and measured values for the parameters included in this study. To obtain the table feed speed, the table feed was divided by the gantry rotation time. As a result, the measured table feed speeds under the low, intermediate, and high table feed settings were determined as 26.67, 48.10, and 64.07 mm/s, respectively.

**Table 1:** Nominal preset values and measured values for the parameters considered in this study

*Table Feed Speed Setting*	*Table Feed Speed (mm/s)*		*Table Feed (mm/rotation)*		*Gantry Rotation Time (ms)*	
	*Preset*	*Measured*	*Preset*	*Measured*	*Preset*	*Measured*
Low	26.67	26.67	20.0	20.1	750	755
Intermediate	48.00	48.10	36.0	36.3	750	755
High	64.00	64.07	48.0	48.3	750	755

**Figure 4:**
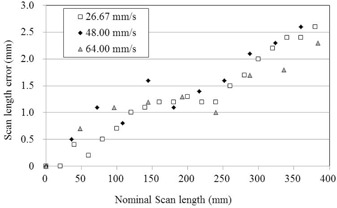
Relation between the nominal scan length and scan length error. For all table feed speed settings, the errors increased linearly as a function of nominal scan length. A scan length of approximately 400 cm would result in an error of over 2 mm. Note that the scan length error is the nominal scan length subtracted from the measured scan length.

It was a simple exercise to measure the distance between the first and subsequent scan peaks derived from the CR cassette, as shown in Fig. 2. After all distances were measured, the nominal distances were subtracted from the measured distances to calculate the scanning location error. Figure 4 shows the relation between the nominal scan length and scan length error for the three table feed speed settings. For all table feed speed settings, the errors increased linearly as a function of nominal scan length.

## IV. DISCUSSION

The table feed speed is an important parameter that CT users have to manipulate when they are setting up scanning protocols. The ratio of scan length to scanning duration is used to determine the slowest table feed required to image the selected anatomical range within the desired time. To make the scanning protocols appropriate, it is important to understand not only the variable setting, but also the accuracy of the table feed speed.

In this study, the CR system and solid‐state detector were employed to measure the table feed and gantry rotation time. The table feed speed was easily obtained by dividing the table feed by the rotation time. We believe that our technique is useful to physicists because 1) the table feed speed and gantry rotation time can be measured without a time‐consuming process, and 2) the technique does not require complicated QA tools or software as the CR system and solid‐state detector are devices familiar to many departments of radiology. The Xi solid‐state detector was employed to determine the gantry rotation time. Moreover, many detectors with a similar temporal resolution are available in the market. Most of these detectors would appear to be applicable for assessing gantry rotation time, although no definite claim can be made without verification.

The table feed speeds for the three table speed settings were measured successfully with an error within 0.1 mm/s. Although the measured values were in excellent agreement with nominal preset values, the results show that measured table feed speeds were faster than nominal settings. On the other hand, the measured gantry rotation time (755 ms) was longer than the nominal setting (750 ms). As a result, scan length error was introduced, as shown in Fig. 4. In this study, the scan length error increase was approximately linear with nominal scan length (0.6%–0.9%). A scanning distance of approximately 400 cm would result in an error of over 2 mm, as shown in Fig. 4. To reduce the error, strict control of table feed speed and gantry rotation time is necessary. Unfortunately, to the best our knowledge, no published studies exist that evaluate the impact of table feed error.

The American College of Radiology published the 2012 CT quality control manual, which includes a test of table travel accuracy.^(12)^ The objective of the test is to verify that the patient table translates in accordance with nominal settings. A phantom with two sets of external fiducial markers of known separation is set on the couch and the distance between the fiducial markers as determined by table travel is compared with the known distance. The ACR test also does not account for potential variable table feed speeds. Therefore, we believe that to maintain the accuracy of table travel, it is important to measure not only the accuracy of the indicator, but also the table feed speed accuracy. The ACR recommends a limit of 2 mm (1.7%) over the 120 mm length of the phantom for its table travel accuracy test. In this study, the measured error over 120 mm scan length was within 1.0 mm (0.8%), which was well within the ACR limits.

It may be necessary that the measurement of table feed speed be conducted with appropriate tabletop loading. AAPM Report 83 recommends that scanner table tests be conducted with the tabletop loaded with at least 150 lb (75 kg) of distributed weight to simulate a patient.^(7)^ In this study, the measurements of the table feed speed were performed without tabletop loading, but the same methodology could be performed with table loading, if desired.

Note that this study has two limitations. First, we had no access to a state‐of‐the art CT system having a high table feed speed of up to 430 mm/s. Under these high‐speed conditions, it would be difficult to measure the table feed speed accurately using the CR system (354 mm×430 mm) employed. A long‐view CR cassette and stitching software may be a promising technique to overcome this limitation.

Second, we determined the radiation beam profile at 80 kVp using a CR cassette in accordance with the recommendation of Liu et al.^(9)^ However, the CR photostimulable phosphor plate is not an adequate replacement for radiographic films to measure the CT beam profile at 120 kVp because the radiation dose is significantly high to delineate a clear radiation profile in “fixed EDR” mode with latitude=4 and sensitivity=5. Many pixels become saturated with the use of such high peak voltages even if the tube current is at its lowest setting (10 mA). Theoretically, there could be differences in the actual beam width of the radiation profile produced at 120 and 80 kVp; however, we believe that the scan length per rotation is invariant.

## V. CONCLUSIONS

Despite some limitations, we proposed a simple noninvasive method for the measurement of table feed speed in modern commercial CT systems. We showed that measurements of CT scanner table feed speed can be accomplished with a CR system and solid‐state detector. In addition, it is noteworthy to point out that the measurement results of table feed speed are in excellent agreement with the nominal preset values. However, the measured gantry rotation time in this study is higher than the nominal setting by 5 ms. Slower gantry rotation caused a scan length error that increased linearly as a function of the nominal scanning length.

Finally, the data suggest that clinical medical physicists should consider adding periodic assessment of the accuracy and precision of the table feed speed to their QA program and consider this in their QC testing, where and when applicable.

## Supporting information

Supplementary Material
