# Supplementary material for: Measurement of table feed speed in modern CT
Source: J Appl Clin Med Phys. 2014 May 8;15(3):275–81. doi: 10.1120/jacmp.v15i3.4703 (PMC5711061; doi:10.1120/jacmp.v15i3.4703)
Supplement: Supplementary file 1 — Supplementary Material [file ACM2-15-275-s001.docx]

Measurement of Table Feed Speed in Modern CT

**Atsushi Fukuda ^1,2^, Pei-Jan Paul Lin ^3^, Kosuke Matsubara ^2^, Tosiaki Miyati ^2^**

*Department of Radiology, Shiga Medical Center for Children, Moriyam city, Shiga, Japan ^1^*

*Division of Health Sciences, Kanazawa University, Graduate School of Medical Sciences,^2^Kodatsuno, Kanazawa, Ishikawa, Japan ^2^*

*Department of Radiology, Virginia Commonwealth University Medical Center, Richmond, Virginia, 23284 ^3^*

*Coresponding author*

*Atsushi Fukuda, MS.*

*Department of Radiology*

*Shiga Medical Center for Children*

*5-7-30, Moriyama, Moriyama city, Shiga prefecture, JAPAN, 524-0022*

*e-mail:* [*ntoki@blue.plala.or.jp*](mailto:ntoki@blue.plala.or.jp)

Running title

Fukuda et al.: Measurement of Table Feed Speed in CT
